# The influence of different types of translational inaccuracies on the genetic code structure

**DOI:** 10.1186/s12859-019-2661-4

**Published:** 2019-03-06

**Authors:** Paweł BłaŻej, Małgorzata Wnetrzak, Dorota Mackiewicz, Paweł Mackiewicz

**Affiliations:** Department of Genomics, University of Wrocław, ul. Joliot-Curie 14a, Wrocław, 50-383 Poland

**Keywords:** Amino acid, Codon, Evolution, Evolutionary algorithm, Graph theory, Optimization, The standard genetic code

## Abstract

**Background:**

The standard genetic code is a recipe for assigning unambiguously 21 labels, i.e. amino acids and stop translation signal, to 64 codons. However, at early stages of the translational machinery development, the codons did not have to be read unambiguously and the early genetic codes could have contained some ambiguous assignments of codons to amino acids. Therefore, the goal of this work was to obtain the genetic code structures which could have evolved assuming different types of inaccuracy of the translational machinery starting from unambiguous assignments of codons to amino acids.

**Results:**

We developed a theoretical model assuming that the level of uncertainty of codon assignments can gradually decrease during the simulations. Since it is postulated that the standard code has evolved to be robust against point mutations and mistranslations, we developed three simulation scenarios assuming that such errors can influence one, two or three codon positions. The simulated codes were selected using the evolutionary algorithm methodology to decrease coding ambiguity and increase their robustness against mistranslation.

**Conclusions:**

The results indicate that the typical codon block structure of the genetic code could have evolved to decrease the ambiguity of amino acid to codon assignments and to increase the fidelity of reading the genetic information. However, the robustness to errors was not the decisive factor that influenced the genetic code evolution because it is possible to find theoretical codes that minimize the reading errors better than the standard genetic code.

## Background

The standard genetic code (SGC) is a template according to which the information stored in a DNA molecule is transmitted to the protein world in the process called translation. This coding system is nearly universal, with some rare exceptions, for almost all living organisms on Earth. The investigations of the unique organization and properties of this code have been carried out ever since the first encoding rules were determined [1, 2]. Many hypotheses were developed to explain the origin and evolution of the SGC (see for review: [3–7]). However, it is still unclear which factor had the decisive impact on its present structure because the results so far are inconclusive and do not allow us to formulate a final explanatory theory [8]. One of the popular hypotheses assumes that the SGC structure has evolved to minimize harmful consequences of mutations or mistranslations of coded proteins [9–24]. Originally, it was assumed that the optimality of the SGC was directly selected.

However, other models of the genetic code evolution were also proposed. In one of such simulation models both the code and the coded message (i.e. genes) could coevolve [25]. The simulations resulted in the codes that were substantially, but not optimally, error-correcting and reproduced the error-correcting patterns of the SGC. In another model, an important role was assigned to horizontal gene transfer, which made the code not only universal and compatible between translational machineries but also optimal [26]. The self-referential model for the formation of the SGC assumes that peptides and RNAs coevolved and were mutual stimulators for the whole system [27]. In this model, a big role was played by tRNA dimers, which directed the initial protein synthesis and showed peptidyl-transferase activity in creation of peptide bonds.

The models assuming a gradual addition of amino acids to the code postulated that this incorporation was: (i) associated with the minimization of disturbance in already synthesized proteins [28], (ii) favoured to promote the diversity of amino acids in proteins [5, 8, 28, 29], (iii) initially driven by catalytic propensity of amino acids functioning in ribozymes [30], (iv) proceeded according to biosynthetic pathways [31–40], or (v) a consequence of duplications of genes coding for tRNAs and aminoacyl-tRNA synthetases (aaRS) [6, 8, 41–47]. The latter proposition, however, was recently criticized in favour of the coevolution theory assuming that the structure of the genetic code was determined by biosynthetic relationships between amino acids [48], although other authors believe that there was a coevolution between the aaRS and the anticodon code as well as an operational code [49]. Thus, the coevolution theory does not necessarily discard the proposition that aaRS and tRNAs played a major role in the formation of the SGC [39].

Considering many factors together, the evolution of the code was probably a combination of adaptation and frozen accident, although contributions of metabolic pathways and weak affinities between amino acids and nucleotide triplets cannot be ruled out [50, 51].

The optimality of the SGC can be reformulated as an attractive problem from the computational and mathematical points of view. For example, a general method of constructing error-correcting binary group codes, represented by channels transmitting binary information, was proposed [52]. Moreover, the analysis of the structure and symmetry of the genetic code using binary dichotomy algorithms also showed its immunity to noise in terms of error-detection and error-correction [53–55]. The code can be also described as a single- or multi-objective optimization problem using the Evolutionary Algorithms (EA) technique to find optimal genetic codes under various criteria [11, 50, 56–58]. Such approach revealed that it is possible to find the theoretical codes much better optimized than the SGC.

The properties of the genetic code can be also tested using techniques borrowed from graph theory [59, 60]. The analysis of the SGC as a partition of an undirected and unweighted graph showed that the majority of codon blocks are optimal in terms of the conductance measure, which is the ratio of non-synonymous substitutions between the codons in this group to all possible single nucleotide substitutions affecting these codons [60]. Therefore, this parameter can be interpreted as a measure of robustness against the potential changes in protein-coding sequences generated by point mutations. The SGC turned out to be far from the optimum according to the conductance but many codon groups in this code reached the minimum conductance for their size [60].

The unique features of the SGC indicate that the structure of this coding system is not fully random and must have evolved under some mechanisms. It is obvious that if we assume that 64 codons encode 20 amino acids and stop coding signal in a potential genetic code then this code must be redundant, i.e. there must exist an amino acid which is encoded by more than one codon. In consequence, such code can be represented as a partition of the set of 64 codons into 21 disjoint subsets (codon groups) so that each codon group encodes unambiguously a respective amino acid or stop signal. Interestingly, these codon groups are generally characterized by a very specific structure in the SGC, namely, the codons belonging to the same group differ usually in the same codon position. Most often the third codon position is different, whereas the first and the second ones stay the same. To explain this specific pattern, Crick developed the wobble rule, which states that the first nucleotide of the tRNA anticodon can interact with one of the several possible nucleotides in the third codon position of a transcript (mRNA) [61]. This non-standard base pairing is often associated with the post-transcriptional modifications of the nucleotide at the first position of the anticodon in the tRNA [62]. The weakened specificity in the base interaction has many consequences. Particularly, it reduces the number of different tRNA molecules which have to recognize codons during the protein synthesis process. Moreover, single point mutations in the third codon position can be synonymous, i.e. do not change the coded amino acid. The wobble base pairing plays also a role in the adoption of the proper structure by tRNA and determines whether the tRNA will be aminoacylated with a specific amino acid.

Our approach to the study of the origins and the possible evolution of the specific structure of the SGC assumes that the early translational machinery was not perfect and codons could be translated ambiguously. Such assumption is in agreement with a hypothesis that protoribosomes could form spontaneously and were able to produce a variety of random peptides, whose sequences depended on the distribution of various amino acids in their vicinity, without the need of a code [63, 64]. Our model also concerns the evolvability of the genetic code as shown in the case of the alternative variants of the genetic code [5, 65–70]. The evolutionary models of these codes postulate the presence of ambiguous assignments of codons to amino acids [71, 72]. Indeed, such assignments were found in Condylostoma, Blastocrithidia and Karyorelict nuclear codes [73–75] as well as *Bacillus subtilis* and *Candida* [76–78]. For these reasons we assumed that the genetic code structure went through intermediate stages in which a particular codon could be translated into more than one amino acid. Obviously, such property of the genetic code is directly related to the level of inaccuracy of the translational machinery. Therefore the goal of our work was to learn which structures of the genetic code can evolve assuming different types of inaccuracy in codon reading in comparison to the structure of the SGC.

Using the approach based on an evolutionary algorithm [79, 80], we analysed a population of randomly generated genetic codes whose codons encoded ambiguously more than one amino acid. The population evolved under the conditions which preferred unambiguous encoding. The scenario which was run under the assumption similar to the wobble rule, produced very quickly the coding systems that are more unambiguous and robust to errors in comparison to other scenarios.

## Methods

In this section we give a brief overview of the technical aspects of our work. First, we set up the notation and the terminology necessary to present the crucial steps of our simulation procedure. Then, we introduce a detailed description of the fitness function *F*, which was used during the selection process. Finally, we describe several measures to study the properties of the optimal genetic codes extracted from the simulations.

### Evolutionary algorithm

To simulate the process of the genetic code emergence, we applied an adapted version of EA class algorithm. This technique is widely used in many optimization tasks, especially in the case when analytical solutions do not exist or they are computationally infeasible [80].

The simulation starts with a population of 1000 candidate solutions (individuals). Each candidate represents a random assignment of 64 codons *c* to 21 labels *l* corresponding to 20 amino acids and stop translation signal. For simplicity of notation, we use the following set of labels *l*=1,2,3,…,20,21 and denote the codons *c*=1,2,3,…,63,64. Therefore, $\mathcal {P}=(p_{cl})$ is a matrix with 64 rows and 21 columns. Each entry *p*_*cl*_ in the matrix $\mathcal {P}$ is a probability that a given codon *c* encodes a given label *l* and every row sums up to one. At the beginning of our simulations, we used the genetic code matrices whose rows were generated according to the uniform distribution. These codes create an unbiased starting population with high volatility.

The simulation process is divided into consecutive steps called generations. During each step, two important operators, i.e. mutation and selection, are applied to the population. The mutation is a classical genetic operator used in all EA algorithms because it is responsible for random modifications of selected individuals, thus creating new solutions. Here this operator is realized by changing the probability that the selected codon encodes one of 21 possible labels. All changes are introduced using random values generated from the normal distribution and normalized to obtain a probability function in each row. The selection operator requires a fitness function *F* which allows for assessing the quality of solutions, i.e. the fitness value. Candidate solutions with greater fitness values (scores) are more likely selected to survive and reproduce for the next generation. In this case, we applied a random process of drawing candidate solutions to the next generation with the probability proportional to their fitness. We run the simulations up to 50,000 steps and repeated them 50 times using different seeds.

### Fitness function

The fitness function *F* plays the decisive role in the procedure of genetic codes selection. As a fitness measure, we used a modified version of the total probability function, i.e. the probability that a given genetic code encodes 20 amino acids and stop translation signal. This measure assumes some restrictions on the structure of the codon group assigned to a specific label, e.g. the size of the potential codon group. Moreover, it favours greater probability of encoding a selected label, which reduces the ambiguity in coding. Below we present a detailed description of *F* in three consecutive steps:

1. Let *L*=*l*_1_,*l*_2_,…,*l*_21_ be a sequence of all labels and let $C=c_{r_{1}}, c_{r_{2}}, \ldots, c_{r_{21}}$, *r*_*i*_=1,2,…,64 be a sequence of random codons where every codon $c_{r_{i}}$ encodes a respective label *l*_*i*_. Each codon $c_{r_{i}} \in C$ is drawn randomly from the set of all possible codons *c*=*c*_1_,*c*_2_,…,*c*_64_ according to the following probability:
  1 $$  P \left(c_{r_{i}}=c_{j} \right)=P\left(c_{j}|l_{i}\right)=\frac{P\left(l_{i}|c_{j}\right)}{\sum_{j=1}^{64}P \left(l_{i}|c_{j} \right)},  $$
  where $p\left (l_{i} | c_{j}\right)=p_{l_{i} c_{j}}$ is an element from $l_{i}^{th}$-row and $c_{j}^{th}$-column of the matrix $\mathcal {P}$. It is evident that $ \sum _{j=1}^{64}P(l_{i}|c_{j}) $ is a sum of all elements extracted from the column *l*_*i*_ of the matrix ${\mathcal {P}}$. Therefore, the Eq. (1) is clearly an application of Bayes rule under the assumption that *a* *priori* probability, i.e. the probability of choosing a given codon *c*_*j*_, is uniformly distributed i.e. *P*(*c*_*j*_)=1/64.
2. For each codon $c_{r_{i}}$ belonging to *C*, we define a codon neighbourhood $N\left (c_{r_{i}}\right)$. $N\left (c_{r_{i}}\right)$ is a set of codons that contains the original codon $c_{r_{i}}$ and the codons $c^{\prime }_{r_{i}}$ differing in one nucleotide from $c_{r_{i}}$. The size of $N\left (c_{r_{i}}\right)$ depends on the simulation assumptions. We considered three possible scenarios:
  *M*_1_ - all codons belonging to a given $N\left (c_{r_{i}}\right)$ have two fixed codon positions identical and differ in exactly one nucleotide at the other position in codon;
  *M*_2_ - all codons belonging to a given $N\left (c_{r_{i}}\right)$ have one fixed codon position identical and differ in exactly one nucleotide in one of the other two codon positions;
  *M*_3_ - all codons belonging to a given $N\left (c_{r_{i}}\right)$ differ in exactly one nucleotide in any codon position.
  For example, the neighbourhood for the codon GGG is:
  GGG, GGA, GGC, GGT for the scenario *M*_1_;
  GGG, AGG, CGG, TGG, GAG, GCG, GTG for the scenario *M*_2_;
  GGG, AGG, CGG, TGG, GAG, GCG, GTG, GGA, GGC, GGT for the scenario *M*_3_.
  Thus, the size of the neighbourhood for *M*_1_ is |*N*(*c*_*r*_)|=4, for *M*_2_ is |*N*(*c*_*r*_)|=7 and for *M*_3_ is |*N*(*c*_*r*_)|=10.
3. Using the assumptions presented in step 1 and 2, we can define the fitness function *F* as:
  2 $$  F=\sum_{c^{\prime}_{r_{1}}, \ldots, c^{\prime}_{r_{21}}: \ c^{\prime}_{r_{i}} \in N\left(c_{r_{i}}\right)} P\left(l_{1}|c^{\prime}_{r_{1}}\right) P\left(l_{2}|c^{\prime}_{r_{2}}\right) \cdot \ldots \cdot P\left(l_{21}|c^{\prime}_{r_{21}}\right) \ .  $$
  It is evident that assuming $P\left (c^{\prime }_{r_{i}}\right)=\frac {1}{64},\ c^{\prime }_{r_{i}}=1,2,\ldots, 64$ and the independence of $P\left (l_{n}|c^{\prime }_{r_{i}}\right)$ in the formula (2), we obtain the following equality:
  $$P(l_{1},l_{2},\ldots,l_{21})=F\cdot \left(\frac{1}{64}\right)^{21}\, $$
  which is the total probability that a given genetic code generates a sequence of labels *L*. Therefore, a high value of *F* suggests that a given genetic code is more likely to encode 20 amino acids and stop coding signal unambiguously.

It should be noted that the computation of *F*, using the formula (2) directly, involves the order of *O*(|*N*(*c*_*r*_)|^21^) calculations [81]. Therefore, fast calculation of the fitness values for many candidate solutions becomes a problem because the “direct” method is computationally infeasible even for small sizes of *N*(*c*_*r*_). To deal with it, we incorporated a modified version of the forward algorithm [81], which is more efficient in computing the exact fitness values than the direct approach. This procedure follows from some basic observations. Let us consider *α*_*l*_(*c*) defined inductively as:

$$\begin{aligned} \alpha_{l}(c)\,=\, \left\{\! \begin{array}{lc} \alpha_{1}(c) \,=\, P(l_{1}|c), & c\in N(c_{r_{1}})\\ \alpha_{k}(c) \,=\, \sum_{c^{\prime}\in N\left(c_{r_{k-1}}\right)}\!\alpha_{k-1}\left(c^{\prime}\right)\!\cdot \!P(l_{k}|c), & 1\!< \!k \leq 21, c\in N(c_{r_{k}}).\\ \end{array}\right. \end{aligned} $$

From this definition we can deduce that $F=\sum _{c\in N\left (c_{r_{21}}\right) }\alpha _{21}(c)$. If we take into account the computational effort required to calculate $\phantom {\dot {i}\!}\alpha _{l}(c)\ c\in N(c_{r_{l}})$ and then compute the fitness value, we need the order of $O\left (|N(c_{r_{l}})|^{2}\right)$ calculations. Thereby, assuming that $\left |N\left (c_{r_{l}}\right)\right |=10$, which is the maximum size of the codon neighbourhood in the *M*_3_ model, we need about 2100 computations for the modified forward method in comparison to about 10^21^ computations for the “direct” approach. This forward procedure allowed us to calculate the fitness values fast and effectively, which is essential in the case of many individuals constantly modified during simulations.

**Fig. 1:**
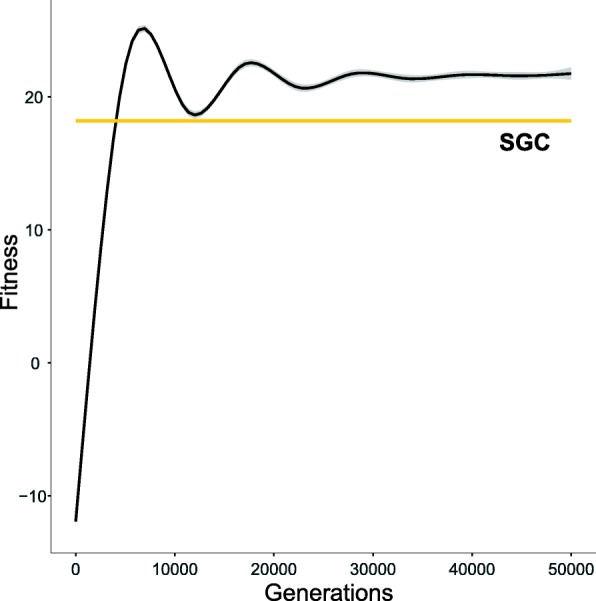
Changes in the best approximation of the fitness function *F* with the number of generations (the black line). All approximations were done for 50 simulations using the Generalized Additive Models. The simulations were run under *M*_1_ scenario with different initial seeds. The independent simulations show a very narrow confidence interval depicted by the grey strip. The results were compared with the average fitness value calculated for the standard genetic code (the orange line)

There is also another important feature related to the fitness function, namely, *F* is non-deterministic. This is because the fitness value is dependent on a randomly generated codon sequence *C*. Therefore, *F* is a random variable and in consequence, genetic codes are rated according to their randomly generated fitness values during the selection process. However, the chance to be selected to the next generation is not only a matter of luck because the selection of the sequence *C* prefers the codons that have relatively high probabilities to encode respective labels (see Eq. (1)). Thereby, the distribution of *F* prefers larger values. They are compared during the selection process and finally, the method of codon selection is crucial in terms of the convergence of genetic codes to the stable solutions. We observed such convergence of the fitness values to the stable solution during the simulations steps. An example of the variation in the fitness function values calculated for 50 independent simulations under the same parameters but different seeds is presented in the Fig. 1.

### Measures of the properties of genetic codes

Because of the large amount of data to analyse, we introduced some definitions to test in details the properties of the obtained genetic codes. One of the most important questions which arose in our investigations was how to measure the level of the genetic code ambiguity at the global scale, because the fitness function delivered us only a piece of information about the probability of encoding 21 labels. To test the quality of a given genetic code, we defined the genetic code entropy.

#### **Definition 1**

Let $\mathcal {P}=\left (p_{cl}\right)$ be a matrix of a genetic code, where each row contains a discrete probability distribution, then the entropy of the genetic code $H(\mathcal {P})$ is defined as:

3 $$ H(\mathcal{P}) = - \sum\limits_{c=1}^{64} \sum\limits_{l=1}^{21} p_{cl} log(p_{cl})\ .  $$

It should be noted that $H(\mathcal {P})$ is in fact the sum of Shannon entropy calculated for each row of the matrix $\mathcal {P}$, separately. Therefore, $H(\mathcal {P})$ corresponds to the multidimensional entropy of independent distributions. The definition 1 appears useful in testing the general properties of genetic codes in terms of changes in their ambiguity. Moreover, it allows us to make more detailed comparisons between the results obtained under different scenarios i.e. *M*_1_,*M*_2_ and *M*_3_. In our analyses we also calculated the average genetic code entropy value $H_{av}(\mathcal {P})$, which is the arithmetic mean of the genetic code entropy $H(\mathcal {P})$ evaluated for all candidate solutions.

Furthermore, we used a graph representation of the genetic code. This approach was effectively applied by [59] and [60]. The authors considered a graph *G*(*V,E*) with 64 nodes (codons) *V* and the set of edges *E* representing point mutations between codons. According to this approach, every genetic code $\mathcal {C}$ is a partition of *V* into 21 disjoint subsets *S*_*l*_, *l*=*l*_1_,*l*_2_,…,*l*_21_, i.e. groups of codons. To investigate further the properties of a given graph clustering, [60] introduced the set conductance, which turned out a very useful measure in testing the properties of codon groups. The definition of the set conductance is as follows:

#### **Definition 2**

For a given graph *G*, let *S* be a subset of *V*. The conductance of *S* is defined as:

$$\phi(S)=\frac{E\left(S,\bar{S}\right)}{vol(S)} \, $$

where $E\left (S,\bar {S}\right)$ is the number of edges of *G* crossing from *S* to its complement $\bar {S}$ and *vol*(*S*) is the sum of all degrees of the vertices belonging to *S*.

The set conductance has a useful interpretation from the biological point of view because for a given codon group *S*, *ϕ*(*S*) is the ratio of non-synonymous codon changes to all possible changes concerning all codons belonging to this set. Therefore, it is interesting to find the optimal codon blocks in terms of *ϕ*(*S*). To do so, we used the *k*-size-conductance *ϕ*_*k*_(*G*) described as the minimal set conductance over all subsets of *V* with the fixed size *k*.

#### **Definition 3**

The *k*-size-conductance of the graph *G*, for *k*≥1, is defined as:

$$\phi_{k} (G) = {min}_{S\subseteq V,|S|=k} \phi(S)\ . $$

Moreover, the properties of a given genetic code $\mathcal {C}$ can be expressed as the average code conductance $\Phi ({\mathcal {C}})$, which is the arithmetic mean calculated from all set conductances of all codon groups. The detailed definition of the average code conductance is given in the following way:

#### **Definition 4**

The average conductance of a genetic code $\mathcal {C}$ is defined as:

$$\Phi(\mathcal{C})=\frac{1}{21}\sum_{S\in\mathcal{C}}\phi(S) \ . $$

### The relationship between matrix and graph representation of the genetic code

As mentioned in the previous section, we used two different representations of the genetic code. The first one describes the genetic codes as a matrix, whereas the other one presents the genetic code as a partition of graph nodes into 21 non-empty disjoint clusters. It is evident that for every graph representation we can construct directly a unique matrix. Then, each row *c* of the matrix $\mathcal {P}$ contains a degenerated probability distribution, i.e. *p*_*cl*_=1, where a codon *c* encodes a label *l*. On the other hand, without additional assumptions, it is impossible to obtain a unique graph partition from a selected matrix representation. Therefore, we have to assume that each row of the matrix $\mathcal {P}$ contains a unimodal probability distribution. Only in such case we can transform $\mathcal {P}$ unambiguously into an equivalent graph representation. To do so, we introduced the maximum likelihood graph partition (MLGP) approach.

#### **Definition 5**

Let $\mathcal {P}=(p_{cl})$ be a matrix representation of a genetic code, where each row contains a unimodal discrete probability distribution. Assume also that for every label *l* there exists a codon *c* such that:

$$p_{cl}={max}_{1\leq l^{\prime}\leq 21}p_{cl^{\prime}}\ . $$

Then the maximum likelihood graph partition is a partition of the set of the graph *G* nodes into 21 non-empty disjoint subsets *S*_1_,*S*_2_,…,*S*_21_ according to the following formula:

$$c\in S_{l} \iff p_{cl}={max}_{1\leq l^{\prime}\leq 21}p_{cl^{\prime}}\ . $$

To measure the quality of the selected codon block *S*_*l*_, *l*=1,2,…,21, created according to the definition 5, we defined the coding strength of the set *S*_*l*_.

#### **Definition 6**

Let ${\mathcal {P}}=(p_{cl})$ be a matrix representation of a genetic code, where each row contains a unimodal discrete probability distribution and let $\mathcal {C}=\{S_{1},S_{2},\ldots S_{21}\}$ be its respective MLGP representation, then for every *S*_*l*_ we define *ψ*(*S*_*l*_), the coding strength of the set *S*_*l*_, in the following way:

$$\psi(S_{l})=\frac{1}{|S_{l}|}\sum\limits_{c\in S_{l}}p_{cl}\ . $$

Following the definition 6 of the coding strength, we can also consider the average coding strength $\Psi (\mathcal {C})$ of a genetic code $\mathcal {C}$, which is defined as the arithmetic mean of all coding strengths *ψ*(*S*_*l*_) computed for all *S*_*l*_ belonging to the graph representation of a genetic code $\mathcal {C}$:

$$\Psi(\mathcal{C}) = \frac{1}{21} \sum\limits_{l=1}^{21}\psi(S_{l})\ . $$

## Results

### The uncertainty level of simulated genetic codes

**Fig. 2:**
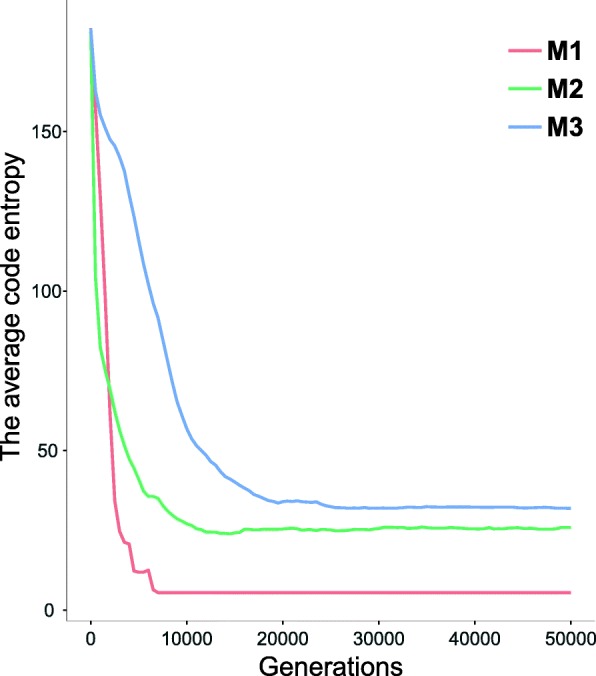
Changes in the average genetic code entropy value $H_{av}(\mathcal {P})$ during the simulation time calculated for three scenarios *M*_1_,*M*_2_,*M*_3_. The average genetic code entropy is the arithmetic mean of the genetic code entropy $H(\mathcal {P})$ evaluated for all candidate solution

The aim of these simulations was to learn, which structures of the genetic codes can evolve assuming different inaccuracy of the translational machinery. We simulated three scenarios of the genetic code evolution that started from an ambiguous coding state. The scenarios *M*_1_,*M*_2_ and *M*_3_ assumed that respectively one, two or three codon positions can be mutated or erroneously read during the translation process. We started our analysis by looking at the differences between the average entropy value $H_{av}(\mathcal {P})$ of the genetic codes calculated for the three scenarios. The high value of the entropy means that a code is characterized by a high level of coding ambiguity, i.e. a individual codon can be translated into various amino acids, while the low values indicate that the coding is more unambiguous. The code with the perfect unambiguity should be characterized by $H_{av}(\mathcal {P}) = 0$. The changes in the coding ambiguity during the simulation time are presented in the Fig. 2 for all types of scenarios. It is evident that $H_{av}(\mathcal {P})$ decreases substantially from the beginning of the simulations under all scenarios and then stabilizes around 10,000 to 30,000 simulation steps. This result indicates that the assumptions used in the optimization procedure are generally responsible for decreasing the uncertainty level of genetic codes. In addition, the level of $H_{av}(\mathcal {P})$ differs between the scenarios. The less extensive the neighbourhood, i.e. the number of similar codons in the group, the smaller the entropy. Under the *M*_1_ scenario, where the neighbourhood size |*N*(*c*_*r*_)|=4, the entropy is the smallest, i.e. 5.48 and the equilibrium is reached much faster than in the other models. The value of $H_{av}(\mathcal {P})$ decreased about 33 times in comparison to the initially ambiguous codes with $H_{av}(\mathcal {P}) \approx 182$. On the other hand, the simulation run under the *M*_3_ scenario, where the neighbourhood is the largest, i.e. |*N*(*c*_*r*_)|=10, reaches its minimum of the $H_{av}(\mathcal {P})$ much later. The entropy of the *M*_3_ scenario is the largest of all scenarios and is almost six times greater than the entropy of *M*_1_ (Fig. 2).

**Fig. 3:**
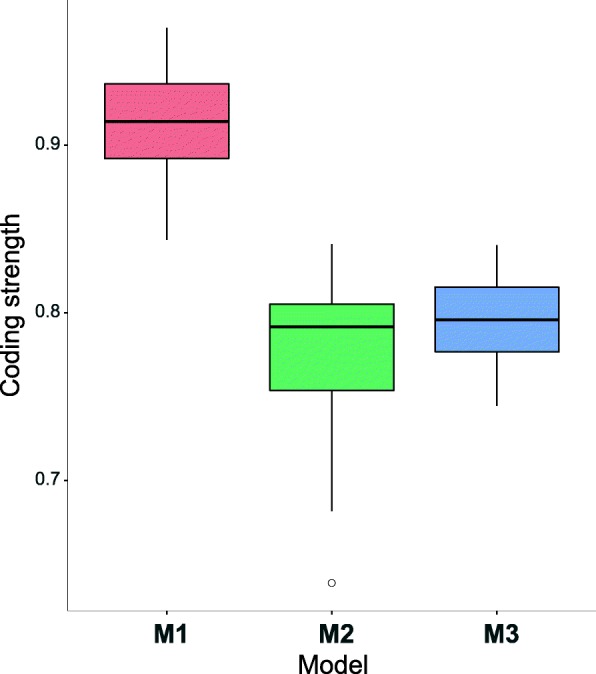
Box-plots of the average coding signal strength calculated at the end of the simulations under three scenarios *M*_1_,*M*_2_ and *M*_3_ for 50 independent simulation runs per scenario. The thick horizontal line indicates the median (*IQR*, the inter-quartile range), the box shows the range between the first and the third quartiles and the whiskers determine the range without outliers for the assumption 1.5×*IQR*

In contrast to the entropy measure, which includes in the calculation the probabilities of all possible assignments of amino acids to codons, the average coding strength *Ψ* takes into account only the maximum probability of these assignments. Large values of *Ψ* indicate that the assignments are highly unambiguous in a given code, while small values mean that many amino acids can be encoded by many codons with a comparable probability. The code with no ambiguous assignment of amino acids to codons ought to have the value *Ψ*=1. Similarly to the entropy, the highest unambiguity and the largest values of *Ψ* are observed in the case of *M*_1_ but the values of *Ψ* do not show the relationship with the size of *N*(*c*_*r*_) as the $H_{av}(\mathcal {P})$ (Fig. 3). We could expect that a decrease in the neighbourhood would result in an increase of the coding signal. However, it is not fully fulfilled because *Ψ* for *M*_2_ is slightly smaller than for *M*_3_ (Fig. 3). This observation suggests that the MGLP graph representations of the genetic codes computed under the *M*_2_ scenario are composed of codon blocks characterized by a weaker coding signal in comparison to the other simulation scenarios.

### The robustness level of simulated genetic codes

**Fig. 4:**
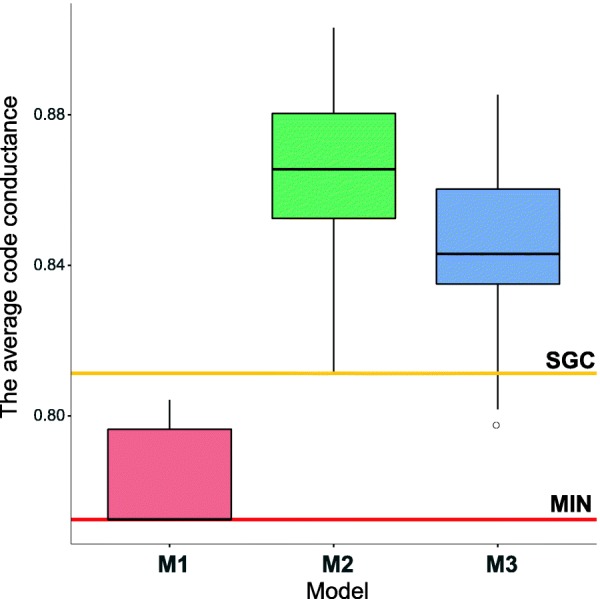
Box-plots of the average code conductance calculated at the end of the simulations under three scenarios *M*_1_,*M*_2_ and *M*_3_ for 50 independent simulation runs per scenario. The thick black horizontal line (inside each box) indicates the median (*IQR*, the inter-quartile range), the box shows the range between the first and the third quartiles and the whiskers determine the range without outliers for the assumption 1.5×*IQR*. The results were compared with the average code conductance *Φ* calculated for the standard genetic code (the orange horizontal line) and the minimum value of the average code conductance (the red horizontal line)

To describe the robustness of the structure of the genetic code to mutations and mistranslations, we applied the average code conductance *Φ*. Its large value indicates that the code is not robust against point mutations. The *Φ* values were calculated following the MLGP representation of the codes obtained at the end of each simulation run. It is interesting that the *Φ* values for each simulation run under the *M*_1_ assumption, are smaller than the average code conductance computed for the standard genetic code, i.e. *Φ*(*SGC*)=0.8112 (Fig. 4). Moreover, the *M*_1_-type optimal genetic codes are closer to the best (minimum) possible value of *Ψ*=0.7724 for any code assigning 21 labels to 64 codons. The results strongly suggest that the *M*_1_ scenario of code evolution is able to create the genetic codes quite robust to mutation and mistranslations. In contrast to that, the genetic codes obtained under the *M*_2_ and *M*_3_ assumptions are characterized by much larger values of the average code conductance than SGC (Fig. 4). Thereby their structures are less robust against point mutation. The genetic codes obtained in the *M*_2_ type of simulations show generally the worst *Φ* in comparison to the other simulation types.

### The types of codon groups in simulated genetic codes

**Fig. 5:**
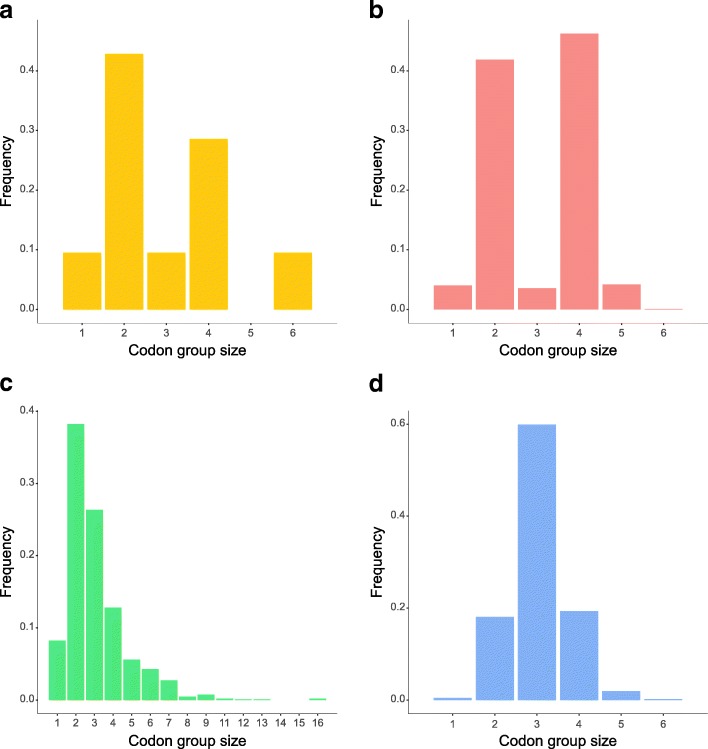
The frequencies of codon group sizes observed in the standard genetic code (**a**) as well as in the MLGP representations of genetic codes at the end of 50 independent simulation runs under the *M*_1_ (**b**), *M*_2_ (**c**) and *M*_3_ (**d**) scenarios

The genetic codes obtained under *M*_1_,*M*_2_ and *M*_3_ scenarios differ in the codon group distribution (Fig. 5). In the the genetic codes produced at the end of 50 independent simulations in the *M*_1_ scenario, there are two most frequent types of groups, consisting of two and four codons (Fig. 5b), similarly to the SGC (Fig. 5a). They constitute in total over 87% of all codon groups in the *M*_1_ codes and 71% in the case of the SGC. The groups of one, three, five and six codons are in the minority, constituting in total less than 13% of the codon groups in the *M*_1_ codes. However, there are also some differences in comparison to the SGC. In the SGC the contribution of two-codon groups is greater than the four-codon groups, while in the *M*_1_ codes the opposite is true. Moreover, there are no groups of five codons in the SGC, which occur in the *M*_1_ codes.

The codes produced by the *M*_2_ model show definitely different distribution of the codon groups and are characterized by a greater variability in codon group sizes, being in the range from 1 to 16 (Fig. 5c). However, the codon groups of the size from 1 to 6 have the joint frequency over 95%. The most frequent are two-codon groups as in the SGC. They constitute 38% and 43%, respectively. What is more, an intriguing kind of symmetry is present in the distribution of codon groups in the genetic codes simulated under the *M*_3_ scenario (Fig. 5d). The most frequently observed codon group consists of three codons and constitutes about 60% of all groups. The frequencies of other codon groups are nearly symmetrically arranged around the most frequent group. The next most common groups (about 20%) include two and four codons. This type of codes are the most different form the SGC in the distribution of the codon groups because in the SGC the three-codon groups are poorly represented.

The presence of codon groups with the number of codons different than in the SGC would seem intriguing and artificial for the simulated codes. However, such groups have actually evolved in some alternative variants of the SGC. In total in these codes, there are five pentacodonic amino acids, four heptacodonic amino acids and five octacodonic amino acids (https://www.ncbi.nlm.nih.gov/Taxonomy/Utils/wprintgc.cgi). For example, in the alternative yeast nuclear code, serine is encoded additionally by the seventh codon CUG, which was taken from leucine, encoded in consequence by five codons.

### The properties of the best genetic codes

**Fig. 6:**
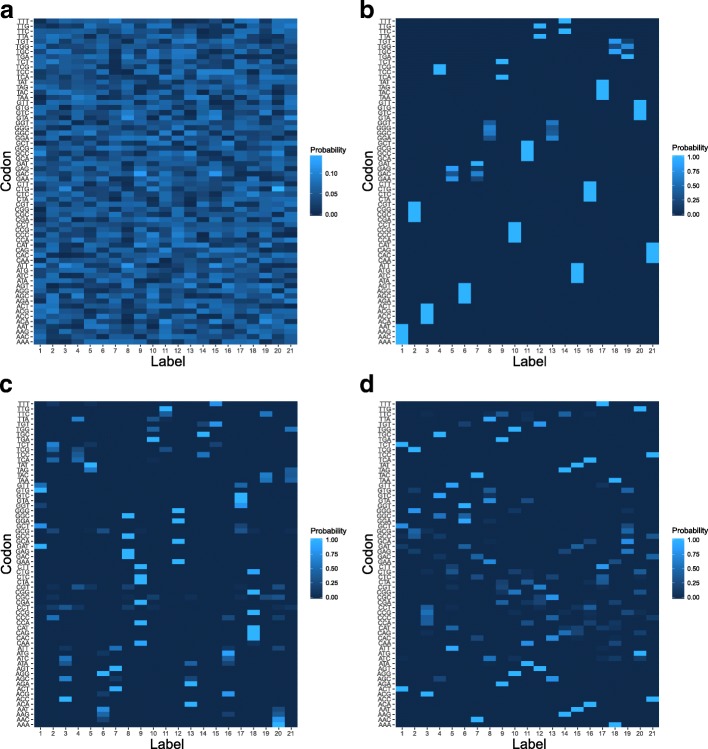
The matrix representation of a genetic code at the beginning of the simulations (**a**) as well as obtained at the end of the simulations under the *M*_1_ (**b**), *M*_2_ (**c**) and *M*_3_ (**d**) scenarios. Each row contains values of the probability function represented by a respective rectangle. The colour of the rectangles indicates high (light blue) or low (dark blue) probability that a given codon (row) encodes a given label (column). It is evident that codon blocks of the size 2 and 4 show high probabilities (light blue colour) and dominate in the code under the *M*_1_ scenario. In the case of other scenarios the codes show much greater ambiguity

In this section, we discussed the properties of the best genetic codes that were selected according to their maximum fitness values from all simulation runs for all types of scenarios. In the Fig. 6, we presented four heatmaps depicting the selected matrix representations of the genetic codes at the beginning as well as at the end of the simulations under the *M*_1_,*M*_2_ and *M*_3_ scenarios.

**Fig. 7:**
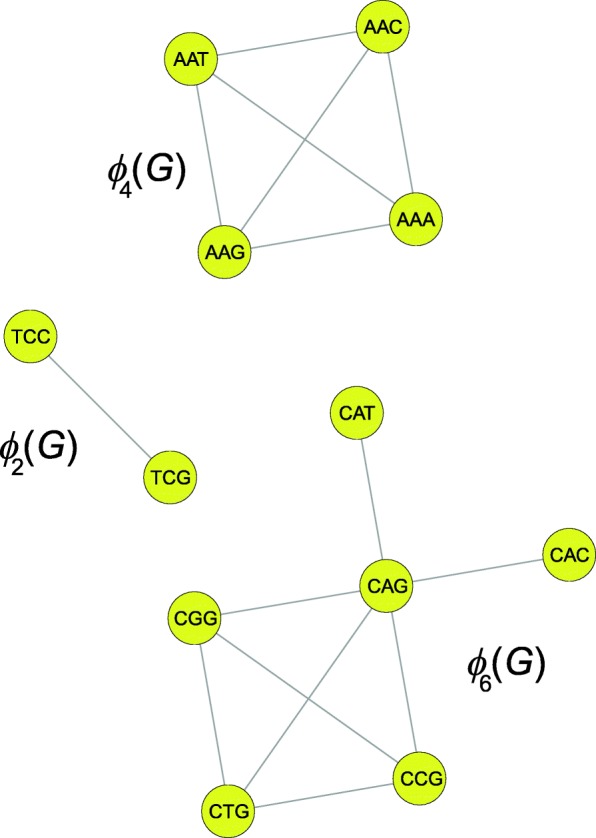
The examples of graph representations of codon groups with the minimal 2, 4 and 6-size conductance: *ϕ*_2_(*G*), *ϕ*_4_(*G*) and *ϕ*_6_(*G*), respectively. The first two cases dominate in the best genetic code produced under the *M*_1_ scenario and the latter is observed in the best genetic code produced under the *M*_2_ scenario

As expected, the random code at the start of simulation is highly ambiguous (Fig. 6a), while the code emerged under the *M*_1_ scenario is characterized by a very high unambiguity and is filled mainly with the codon blocks consisting of two and four codons (Fig. 6b). The codons in each of such groups differ in pairwise comparison in only one nucleotide (Fig. 7). The graph representation of this code following the definition 5 is also optimal in terms of the *k*-size conductance *ϕ*_*k*_(*G*), *k*=2,4. All the codon groups show the minimum possible conductance for their size. Therefore, these groups are the most robust against single non-synonymous nucleotide mutations. In consequence, this genetic code reaches the minimum of the average code conductance $\Phi (\mathcal {C})=0.7725$, which is the minimum value of all possible genetic codes and is smaller than the conductance of the standard genetic code *Φ*(*SGC*)=0.8113. Moreover, many codon groups in the *M*_1_-type code are characterized by a relatively large unambiguity. Fifteen groups have the maximal coding strength *ψ*(*S*)=1 and the average coding strength calculated over all 21 groups is equal to 0.9375 (Table 1).

**Table 1:** The codon groups of the best genetic code in terms of the fitness function *F* extracted from 50 independent simulations under the *M*_1_ scenario

Codon group (*S*)	*k*	*ψ*(*S*)	*ϕ*(*S*)	*ϕ*_*k*_(*G*)
{*AAA,AAT,AAG,AAC*}	4	1.0000000	$\frac {2}{3}$	$\frac {2}{3}$
{*CGA,CGT,CGG,CGC*}	4	1.0000000	$\frac {2}{3}$	$\frac {2}{3}$
{*ACA,ACT,ACG,ACC*}	4	1.0000000	$\frac {2}{3}$	$\frac {2}{3}$
{*GTA,GTT,GTG,GTC*}	4	1.0000000	$\frac {2}{3}$	$\frac {2}{3}$
{*CAA,CAT,CAG,CAC*}	4	1.0000000	$\frac {2}{3}$	$\frac {2}{3}$
{*AGA,AGT,AGG,AGC*}	4	1.0000000	$\frac {2}{3}$	$\frac {2}{3}$
{*CCA,CCT,CCG,CCC*}	4	1.0000000	$\frac {2}{3}$	$\frac {2}{3}$
{*ATA,ATT,ATG,ATC*}	4	1.0000000	$\frac {2}{3}$	$\frac {2}{3}$
{*CTA,CTT,CTG,CTC*}	4	1.0000000	$\frac {2}{3}$	$\frac {2}{3}$
{*TAA,TAT,TAG,TAC*}	4	1.0000000	$\frac {2}{3}$	$\frac {2}{3}$
{*GCA,GCT,GCG,GCC*}	4	1.0000000	$\frac {2}{3}$	$\frac {2}{3}$
{*TCG,TCC*}	2	1.0000000	$\frac {8}{9}$	$\frac {8}{9}$
{*TCA,TCT*}	2	1.0000000	$\frac {8}{9}$	$\frac {8}{9}$
{*TTA TTG*}	2	1.0000000	$\frac {8}{9}$	$\frac {8}{9}$
{*TTT,TTC*}	2	1.0000000	$\frac {8}{9}$	$\frac {8}{9}$
{*TGT,TGC*}	2	0.8648035	$\frac {8}{9}$	$\frac {8}{9}$
{*TGA,TGG*}	2	0.8648030	$\frac {8}{9}$	$\frac {8}{9}$
{*GAT,GAC*}	2	0.8344635	$\frac {8}{9}$	$\frac {8}{9}$
{*GAA,GAG*}	2	0.8344630	$\frac {8}{9}$	$\frac {8}{9}$
{*GGG,GGC*}	2	0.6446255	$\frac {8}{9}$	$\frac {8}{9}$
{*GGA,GGT*}	2	0.6446250	$\frac {8}{9}$	$\frac {8}{9}$

The groups *S* are characterized by: the size *k*, the coding strength *ψ*(*S*), the conductance *ϕ*(*S*) and the minimal conductance of the codon group with the size *k* *ϕ*_*k*_(*G*)

The best codes produced under the *M*_3_ scenario (Fig. 6d) show completely different composition of codon groups in comparison to the best code of the *M*_1_ scenario. The *M*_3_-type code is composed of codon groups of the size *k*=2,3,4 with the domination of three-codon groups (Table 2). This code is also less robust against point mutation because its average code conductance is equal to 0.8457, which is slightly greater than the conductance of the standard genetic code *Φ*(*SGC*)=0.8113. This is caused by the presence of as many as twelve non-optimal codon groups in terms of the k-size conductance (Table 2). The code shows a higher ambiguity than that of the *M*_1_ scenario because its average coding strength *ψ* is 0.8023. Only four codon groups consisting of two codons are perfectly unambiguous and robust to non-synonymous mutations.

**Table 2:** The codon groups of the best genetic code in terms of the fitness function *F* extracted from 50 independent simulations under the *M*_3_ scenario

Codon group (*S*)	*k*	*ψ*(*S*)	*ϕ*(*S*)	*ϕ*_*k*_(*G*)
{*AAG,TAG,TTC,CAG*}	4	0.7453878	$\frac {5}{6}$	$\frac {2}{3}$
{*ATC,TTA,GAA,GTA*}	4	0.7347630	$\frac {8}{9}$	$\frac {2}{3}$
{*ACG,CCA,CCT,CCG*}	4	0.5928058	$\frac {7}{9}$	$\frac {2}{3}$
{*AGC,CAC,CGC,CCC*}	4	0.6837612	$\frac {7}{9}$	$\frac {2}{3}$
{*GAG,GTG,GCA,GCG*}	4	0.5734470	$\frac {7}{9}$	$\frac {2}{3}$
{*ACT,TCT,GCT*}	3	0.9164170	$\frac {7}{9}$	$\frac {7}{9}$
{*AGG,TGG,CGG*}	3	0.8623860	$\frac {7}{9}$	$\frac {7}{9}$
{*TGC,GTC,GGC*}	3	0.8267687	$\frac {23}{27}$	$\frac {7}{9}$
{*AGT,TGT,CGT*}	3	0.8261313	$\frac {7}{9}$	$\frac {7}{9}$
{*ACC,TCC,GAC*}	3	0.8157710	$\frac {25}{27}$	$\frac {7}{9}$
{*ATG,TTG,CTG*}	3	0.7968670	$\frac {7}{9}$	$\frac {7}{9}$
{*GAT,GGA,GGT*}	3	0.7812357	$\frac {23}{27}$	$\frac {7}{9}$
{*ATT,GTT,CAT*}	3	0.7741430	$\frac {25}{27}$	$\frac {7}{9}$
{*TTT,CTT,CTC*}	3	0.7475287	$\frac {23}{27}$	$\frac {7}{9}$
{*AGA,TGA,CGA*}	3	0.7347630	$\frac {7}{9}$	$\frac {7}{9}$
{*ATA,CAA,CTA*}	3	0.7112670	$\frac {23}{27}$	$\frac {7}{9}$
{*TCG,GGG,GCC*}	3	0.7241587	1	$\frac {7}{9}$
{*AAC,TAC*}	2	1.0000000	$\frac {8}{9}$	$\frac {8}{9}$
{*AAT,TAT*}	2	1.0000000	$\frac {8}{9}$	$\frac {8}{9}$
{*ACA,TCA*}	2	1.0000000	$\frac {8}{9}$	$\frac {8}{9}$
{*AAA,TAA*}	2	1.0000000	$\frac {8}{9}$	$\frac {8}{9}$

The groups *S* are characterized by: the size *k*, the coding strength *ψ*(*S*), the conductance *ϕ*(*S*) and the minimal conductance of the codon group with the size *k* *ϕ*_*k*_(*G*)

The best genetic code evaluated under the *M*_2_ model (Fig. 6c) is characterized by the most diversified size of codon groups in comparison to the *M*_1_ and *M*_3_ cases because it is composed of codon groups of the size *k*=1,2,3,4,6 (Table 3). These groups are also characterized by generally smaller coding strength values of *ψ*. Therefore, the average coding strength calculated in this case is equal to 0.7996. Moreover, thirteen codon blocks are not optimal in terms of the set conductance *ϕ*(*S*). In consequence, the average code conductance is relatively high and equals 0.8580. Therefore, it is the least robust genetic code structure against point mutation in comparison to the *M*_1_- and *M*_3_-type codes. The *M*_2_ code contains no codon groups including at least two codons that simultaneously encode unambiguously one label and are the most robust to single point mutations. On the other hand, the two largest groups of six codons in this code are optimal in terms of the *k*-size conductance *ϕ*_*k*_(*G*) (Fig. 7) and are characterized by quite big values of coding strength, over 0.98.

**Table 3:** The codon groups of the best genetic code in terms of the fitness function *F* extracted from 50 independent simulations under the *M*_2_ scenario

Codon group (*S*)	*k*	*ψ*(*S*)	*ϕ*(*S*)	*ϕ*_*k*_(*G*)
{*CAT,CAG,CAC,CTG,CGG,CCG*}	6	0.9867038	$\frac {36}{54}$	$\frac {36}{54}$
{*CAA,CTA,CTT,CTC,CGA,CCA*}	6	0.9866648	$\frac {35}{54}$	$\frac {36}{54}$
{*GAG,GAC,GGC,GCC*}	4	1.0000000	$\frac {7}{9}$	$\frac {2}{3}$
{*GAA,GGA,GGG,GCA*}	4	1.0000000	$\frac {7}{9}$	$\frac {2}{3}$
{*GAT,GTT,GTG,GCT*}	4	0.8262542	$\frac {7}{9}$	$\frac {2}{3}$
{*GTA,GTC,GGT,GCG*}	4	0.7837840	$\frac {34}{36}$	$\frac {2}{3}$
{*ATC,AGC,ACC,CCT*}	4	0.6426162	$\frac {5}{6}$	$\frac {2}{3}$
{*ATT,AGT,ACT*}	3	0.8457703	$\frac {7}{9}$	$\frac {7}{9}$
{*ATA,AGA,ACA*}	3	0.8136870	$\frac {7}{9}$	$\frac {7}{9}$
{*AAT,AAG,AGG*}	3	0.7359857	$\frac {23}{27}$	$\frac {7}{9}$
{*AAA,AAC,CGC*}	3	0.7170777	$\frac {25}{27}$	$\frac {7}{9}$
{*TAA,TAC,TTC*}	3	0.5927813	$\frac {23}{27}$	$\frac {7}{9}$
{*TCT,TCG,CCC*}	3	0.5598730	$\frac {25}{27}$	$\frac {7}{9}$
{*TTA,TCA,CGT*}	3	0.4932700	$\frac {25}{27}$	$\frac {7}{9}$
{*ATG,ACG*}	2	0.8650405	$\frac {8}{9}$	$\frac {8}{9}$
{*TAT,TAG*}	2	0.8027995	$\frac {8}{9}$	$\frac {8}{9}$
{*TTT,TGT*}	2	0.7850055	$\frac {8}{9}$	$\frac {8}{9}$
{*TGC,TCC*}	2	0.7838025	$\frac {8}{9}$	$\frac {8}{9}$
{*TGA*}	1	1.0000000	1	1
{*TTG*}	1	0.9967190	1	1
{*TGG*}	1	0.5734410	1	1

The groups *S* are characterized by: the size *k*, the coding strength *ψ*(*S*), the conductance *ϕ*(*S*) and the minimal conductance of the codon group with the size *k* *ϕ*_*k*_(*G*)

## Discussion

We carried out a simulation study to find out how the structure of the genetic code could have evolved under various types of inaccurate translation of codons to amino acids. The simulations started from the set of ambiguous assignments of amino acids to codons, which evolved into patterns with lower levels of uncertainty. The reduction of ambiguity was driven by a fitness function, which preferred the codes that are characterized by the robustness to incorrect amino acid translation due to point mutations in codons. We developed three theoretical models of the genetic code evolution, *M*_1_, *M*_2_, and *M*_3_, which corresponded to various types and levels of inaccuracy of a primordial translation apparatus.

All the models are in agreement with the ambiguous intermediate mechanism acting in the evolution of the alternative genetic codes [71, 72]. In this case, the codon is translated ambiguously to two different amino acids during the period of reassignment. Such cases of ambiguous translation were reported in different organisms [73–78]. What is more, such ambiguous state can also promote phenotypic diversity and adaptability, e.g. it helps yeasts to cope with more stressful environments [82, 83].

Moreover, the models *M*_1_ and *M*_2_ match the stages of the genetic code evolution postulated by the 2-1-3 model [44, 84] and the four-column theory [28]. They assume that in the beginning of the genetic code evolution the second codon position decided about the encoded amino acids, whereas other positions were not important. Next, the coding specificity occurred in the first codon position and then, to some extent, in the third position.

The initial ambiguity in the assignments of amino acids to codons disappeared the fastest under the *M*_1_ model. The codes generated under this scenario are characterized by the biggest unambiguity of coding and the most effective minimization of mutations changing encoded amino acids or stop translation signal. On the other hand, the genetic codes simulated under the *M*_3_ assumptions maintained the highest level of ambiguity and the *M*_2_-type codes produced the biggest number of amino acid changes due to point mutations in codons.

It is interesting to consider, which of the simulated codes is the most similar to the SGC based on unambiguity, minimization of point mutations and the structure. According to the unambiguity measured by entropy or coding strength, the most similar are the codes obtained under the *M*_1_ scenario. They show almost unambiguous assignments of amino acids to codons. However, they are not perfect. Similarly, the SGC is usually presented as a table with the unambiguous assignments but the translation process is not ideal and some errors can occur. It was estimated that one mistranslation occurs with the rate of 10^−3^ to 10^−6^ per codon [85] or 10^−3^ to 10^−5^ per amino acid [86]. Moreover, errors associated with replication and transcription processes can also change the encoded amino acid. If the initial genetic codes had been characterized by much bigger ambiguity of assignments of amino acids to codons, they would have been quickly eliminated by selection, which resembles the rapid decrease in entropy during the simulation of the *M*_1_ codes. The entropy in other models was also reduced but stabilized at the much larger level. It indicates that the assumption on a imprecise recognition of only one fixed codon position is necessary to reduce the initial ambiguity, which corresponds to the wobble rule characterizing the current process of translation.

In terms of minimization of amino acid replacements resulting from point mutations in codons, measured here by the average conductance, the SGC is placed between the *M*_1_ codes, characterized by the lowest conductance, and the codes from the *M*_2_ and *M*_3_ models. In agreement with our simulation study, other analyses also showed that the SGC is not perfectly optimized in this respect and better codes can be found [11, 44, 50, 57, 58, 87–89]. Therefore, it is possible that the assignments of amino acids to codons occurred in accordance with other mechanisms, while the minimization of mutation errors was adjusted by the direct optimization of the mutational pressure around the established genetic code [90–94]. Moreover, some minimization properties of the SGC could have evolved as a by-product of the duplication of genes for tRNAs and aminoacyl-tRNA synthetases charging similar amino acids [6, 8, 41–47]. It is also possible that new amino acids were added into the code in an order that ensured the minimal disturbance of already synthesized proteins but the code itself was not directly optimized [28].

When we compare the structure of the SGC with the structure of the codes produced by the three models, the standard code is the most similar to the *M*_1_ codes because they are also characterized by the domination of amino acids encoded by the groups of two and four codons. All these codon groups are also optimal in terms of the conductance in both the simulated and the SGC. However, four-codon groups are the most numerous in the *M*_1_ codes, while in the SGC the most frequent are two-codon groups, which dominate also in the *M*_2_ codes. The degeneracy of the SGC is usually associated with the presence of codons encoding the same amino acid and differing in the third codon position. It corresponds to the *M*_1_ model, in which the codons for a given amino acid have two fixed positions identical and one different. However, the SGC contains also two codon groups encoding arginine and leucine, which resemble the codon groups in the *M*_2_ model, where all the codons in the groups have one fixed codon position identical and differ in one nucleotide in one of the other two codon positions. Three codons recognized as the stop translation signal also show this property in the SGC. Therefore, the SGC is a mixture of the *M*_1_ and *M*_2_ models in this respect.

The models *M*_3_ and *M*_2_ can represent the initial stages of evolution when the translational apparatus did not read codons perfectly. Therefore, there was a selection to improve the translation process and to develop a stable form of the genetic code. The fixing of two codon positions, represented by the *M*_1_ model, was crucial and enough to unambiguously encode 20 amino acids and the stop translation signal by 64 codons. The wobble base pairing could be a relic of the initial ambiguity.

Since the SGC turned out to be most similar to the codes evolved under the *M*_1_ and *M*_2_ models, we may assume that at certain stage it could have evolved according to the theories proposed by [44, 84] and [28], which means that in the beginning the translation machinery could have recognised only the second codon position, then the first and the third positions. It would be interesting to combine our models with others or enrich it with other biological assumptions to obtain a more accurate model of the evolution of the standard genetic code.

## Conclusions

The initial evolution of the standard genetic code could have started from imperfect reading of the genetic information associated with ambiguous assignments of amino acids to codons. Then the selection favoured codes that improved the fidelity of the translation process. An important step was the fixation of two codon positions, which generated the typical codon block structure of the genetic code. According to this hypothesis, the wobble base pairing in the third codon position could be a relic of an early ambiguity. However, the selection for the minimization of translational errors could not have been the only factor influencing the genetic code evolution because its current level of optimization is not perfect. The simulated codes outperformed the standard genetic code in the robustness against mistranslations.

## Abbreviations

aaRS: Aminoacyl-tRNA synthetases
EA: Evolutionary Algorithms
mRNA: Messenger RNA
SGC: Standard genetic code
tRNA: Transfer RNA
